# Platelet activation in experimental murine neonatal pulmonary hypertension

**DOI:** 10.14814/phy2.14386

**Published:** 2020-03-12

**Authors:** Pavel Davizon‐Castillo, Ayed Allawzi, Matthew Sorrells, Susan Fisher, Kristina Baltrunaite, Keith Neeves, Eva Nozik‐Grayck, Jorge DiPaola, Cassidy Delaney

**Affiliations:** ^1^ Section of Pediatric Hematology, Oncology, and Bone Marrow Transplant University of Colorado Anschutz Medical Campus Aurora CO USA; ^2^ Section of Pediatric Critical Care and Cardiovascular Pulmonary Research Laboratory University of Colorado Anschutz Medical Campus Aurora CO USA; ^3^ Department of Chemical and Biological Engineering Colorado School of Mines Golden CO USA; ^4^ Section of Neonatology Department of Pediatrics University of Colorado Anschutz Medical Campus Aurora CO USA; ^5^ Department of Bioengineering University of Colorado Anschutz Medical Campus Aurora CO USA; ^6^ Division of Pediatric Hematology Oncology Washington University in St. Louis St. Louis MO USA

**Keywords:** neonate, platelets, pulmonary hypertension, serotonin

## Abstract

Serotonin (5‐HT) contributes to the pathogenesis of experimental neonatal pulmonary hypertension (PH) associated with bronchopulmonary dysplasia (BPD). Platelets are the primary source of circulating 5‐HT and is released upon platelet activation. Platelet transfusions are associated with neonatal mortality and increased rates of BPD. As BPD is often complicated by PH, we tested the hypothesis that circulating platelets are activated and also increased in the lungs of neonatal mice with bleomycin‐induced PH associated with BPD. Newborn wild‐type mice received intraperitoneal bleomycin (3 units/kg) three times weekly for 3 weeks. Platelets from mice with experimental PH exhibited increased adhesion to collagen under flow (at 300 s^−1^ and 1,500 s^−1^) and increased expression of the αIIbβ3 integrin and phosphatidylserine, markers of platelet activation. Platelet‐derived factors 5‐HT and platelet factor 4 were increased in plasma from mice with experimental PH. Pharmacologic blockade of the 5‐HT 2A receptor (5‐HT 2A R) prevents bleomycin‐induced PH and pulmonary vascular remodeling. Here, platelets from mice with bleomycin‐induced PH demonstrate increased 5‐HT 2A R expression providing further evidence of both platelet activation and increased 5‐HT signaling in this model. In addition, bleomycin treatment increased lung platelet accumulation. In summary, platelets are activated, granule factors are released, and are increased in numbers in the lungs of mice with experimental neonatal PH. These results suggest platelet activation and release of platelet‐derived factors may increase vascular tone, promote aberrant angiogenesis, and contribute to the development of neonatal PH.

## Graphical abstract

Our study utilized a murine bleomycin model of pulmonary hypertension (PH) to study the hypothesis that platelets from mice with PH circulate in an activated state and that circulating and lung platelet‐derived factors, as well as the number of platelets in the lungs of neonatal mice with PH, is significantly increased. Through an extensive characterization of the functional status of platelets from bleomycin‐treated mice, we demonstrate that mice with bleomycin‐induced PH exhibit qualitative but not quantitative platelet changes. Circulating platelets from mice with PH exhibit hyperactivity as measured directly by adhesion and aggregation under flow and the presence of activation markers and indirectly by quantification of platelet‐specific proteins in plasma. Interestingly, we have also found that the absolute number of platelets within the lungs of mice with PH is significantly higher all together suggesting that platelets could directly be promoting PH.

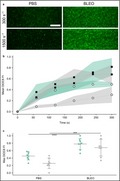

## 1 INTRODUCTION

Pulmonary hypertension (PH) is a life‐threatening condition that develops in 14%–25% of preterm infants with the lung disease of prematurity known as bronchopulmonary dysplasia (BPD) (Bhat, Salas, Foster, Carlo, & Ambalavanan, 2012; Mourani et al., 2015). The pathophysiology of neonatal PH associated with BPD involves alterations in numerous signaling pathways, including the serotonin (5‐HT) pathway (Alvira, 2016; Bhatt et al., 2001; Delaney et al., 2018; Le Cras et al., 1999; Le Cras, Markham, Tuder, Voelkel, & Abman, 2002). We have previously shown that pharmacologic inhibition of 5‐HT signaling via the 5‐HT 2A receptor (5‐HT 2A R) increases pulmonary blood flow in fetal sheep with PH and protects against the development of murine bleomycin‐induced neonatal PH (Delaney et al., 2013, 2018). The vast majority of peripheral 5‐HT (98%) is synthesized by enterochromaffin cells of the small intestine and taken up by platelets via the serotonin transporter (SERT) where it is stored within dense granules (Barter & Pearse, 1953). This mechanism results in plasma levels of 5‐HT in the low nanomolar range while in platelet dense granules, the concentration of 5‐HT reaches the millimolar range (Holmsen & Weiss, 1979). Selective serotonin reuptake inhibitors, via blockade of SERT, increase the plasma 5‐HT levels and have been associated with PH in newborns exposed to these agents during the third trimester of fetal development (Chambers et al., 2006).

Increased plasma 5‐HT and activation of circulating platelets are reported in adults with PH (Damas et al., 2004; Diehl et al., 2011; Herve et al., 1990, 1995; Kazimierczyk & Kaminski, 2018; Kereveur et al., 2000; Nakonechnicov, Gabbasov, Chazova, Popov, & Belenkov, 1996). While no studies have evaluated whether platelets within the lungs of patients who died or received a lung transplant for PH were activated, pulmonary artery thromboses are increased in patients with PH and anti‐platelet therapies targeting the platelet hemostatic response demonstrate clear benefit in patients with chronic thromboembolic PH (Chaouat, Weitzenblum, & Higenbottam, 1996; Moser & Bloor, 1993; Wagenvoort, 1980). Antibody‐induced thrombocytopenia and treatment with pharmacologic platelet inhibitors (aspirin and dipyridamole) protect rats from monocrotaline and hypoxia‐induced PH (Gao et al., 2017; Keith, Will, Huxtable, & Weir, 1987; Mlczoch, Tucker, Weir, Reeves, & Grover, 1978; Shen, Shen, Pu, & He, 2011). Additionally, mice with a platelet‐specific deletion of toll‐like receptor 4 are protected from hypoxia‐induced PH (Bauer et al., 2014). Whether activation of platelets and increased circulating platelet‐derived factors such as 5‐HT are associated with experimental neonatal PH is unknown.

Platelets are small anucleated cells derived from megakaryocytes and are essential for hemostasis. Platelets are also integral mediators of other physiologic processes including immune regulation, vascular inflammation, and wound healing (Golebiewska & Poole, 2015; Kubes, 2016; Opneja, Kapoor, & Stavrou, 2019; Projahn & Koenen, 2012; Rondina & Garraud, 2014; Smyth et al., 2009). Aberrant platelet activation mediates several pathologic conditions in adults including atherosclerosis, sepsis, asthma, and acute lung injury (Lievens & Hundelshausen, 2011; Looney et al., 2009; Middleton, Weyrich, & Zimmerman, 2016; Pitchford, Cleary, Arkless, & Amison, 2019). In neonates, elevated platelet counts after birth are an independent predictor of moderate and severe BPD, which is often associated with PH (Chen, Li, Qiu, Yang, & Walther, 2019). Furthermore, increased plasma platelet‐derived protein, platelet factor 4 (PF4), after birth is associated with higher rates of later pulmonary vascular disease in former preterm infants (Wagner et al., 2018). Interestingly, recent published randomized clinical trials have raised concern about the effect of platelet transfusions on major neonatal outcomes. Preterm neonates transfused with platelets to maintain a higher platelet threshold have higher rates of mortality, BPD, and intraventricular hemorrhage (Curley et al., 2019; Kumar, 2019; Sola‐Visner & Bercovitz, 2016).

The mechanism by which platelets may adversely affect these neonatal outcomes is unknown and raises suspicion for the role of platelet activation in the pathogenesis of other neonatal conditions including PH. Each platelet contains numerous growth factors, vasoactive mediators, chemokines, cytokines, and angiogenic agents that have been implicated in the pathogenesis of PH (Balabanian et al., 2002; Christman et al., 1992; Clave, Maeda, Thomaz, Bydlowski, & Lopes, 2019; Duncan et al., 2012; Flaumenhaft & Sharda, 2019; Hundelshausen, Petersen, & Brandt, 2007; Italiano et al., 2008; Jurasz, Ng, Granton, Courtman, & Stewart, 2010; Kawut et al., 2005; Lopes et al., 2011; Tantawy, Adly, Ismail, Habeeb, & Farouk, 2013). These factors are stored within three types of granules: alpha (PF4 (CXCL4), CXCL7, CXCL5, PDGF, TGF‐ß), dense (5‐HT, Ca, ADP, ATP), and lysosomal (Flaumenhaft & Sharda, 2019). Resting platelets circulate at the margins of blood vessels and are maintained in their resting state primarily by the release of endothelial‐derived mediators such as nitric oxide (NO) and prostacyclin (PGI_2_) (Andrews & Berndt, 2004; Aytekin et al., 2012; Willems & Aken, 1979). With endothelial dysfunction and injury, NO and PGI_2_ release by the endothelium is decreased, platelets adhere to the subendothelial matrix, aggregate and release their granule contents including 5‐HT via exocytosis (Aytekin et al., 2012; Koupenova & Freedman, 2019; Ranchoux et al., 2018). Platelet 5‐HT activates pulmonary vascular receptors increasing pulmonary vascular tone and smooth muscle cell proliferation. In addition, platelet 5‐HT can act in autocrine way by enhancing local platelet activation and aggregation through the platelet 5‐HT 2A R (Mammadova‐Bach, Mauler, Braun, & Duerschmied, 2018).

Whether platelets contribute to the pathogenesis of neonatal PH and are a source of increased circulating mediators known to cause PH is unknown. Our study utilized a murine bleomycin model of PH to study the hypothesis that platelets from mice with PH circulate in an activated state and that circulating and lung platelet‐derived factors, as well as the number of platelets in the lungs of neonatal mice with PH, is significantly increased.

## 2 METHODS

### 2.1 Mouse model

The University of Colorado Denver Institutional Animal Care and Use Committee (IACUC) approved all animal studies. Beginning on days 1–2 of life, C57BL/6 wild‐type mice (Jackson Laboratory) were injected with intraperitoneal phosphate‐buffered saline (PBS) or bleomycin (3 units/kg, dissolved in PBS) (Hospira) three times per week for 3 weeks (total nine injections, 10 μl). This murine injury model of PH and BPD produces similar major pathologic findings to infants with PH and BPD including; impaired alveolar development (decreased radial alveolar counts, increased mean linear intercept and increased air space area), vascular remodeling (decreased vessel density, muscularization of small vessels, medial wall thickening), and PH (right ventricular hypertrophy and elevated right ventricular systolic pressure Delaney et al., 2018; Delaney et al., 2015; Sherlock et al., 2018). Bleomycin doses were adjusted for body weight at each injection. Mice were euthanized for tissue harvesting at 3 weeks of age.

### 2.2 Preparation of mouse blood, platelets, and plasma

Mice were anesthetized with isoflurane and blood was obtained via cardiac puncture of the right ventricle after performing a bilateral thoracotomy using a 21‐gauge needle containing the appropriate anticoagulant (3.8% ACD or heparin). Complete blood counts were obtained within 60 min after blood collection using the veterinarian hematologic analyzer Heska HT5. Platelet‐rich plasma (PRP) was obtained by centrifugation of whole blood at 100 × *g* for 10 min. PRP was supplemented with PGI2 (1 µg/ml) and incubated at room temperature for 3 min prior to centrifugation at 2,000 *g* × 2 min to obtain platelet poor plasma (PPP) or platelet pellets for further washing using PGI2‐containing Tyrodes buffer at 2,000 *g* × 2 min.

### 2.3 Whole blood microfluidic flow assays

Clean glass slides were functionalized with (tridecafluoro‐1,1,2,2tetrahydrooctyl) trichlorosilane (FOTS) via vapor deposition (Mayer, Boer, Shinn, Clews, & Michalske, 2000). Collagen‐related peptides (CRP), integrin α_2_β_1 _ligand (GFOGER), and von Willebrand factor binding peptide (VWF‐BP) were patterned on glass to simulate the major interactions between platelets and type I collagen. These peptides mimic the binding domains on type I collagen for platelets’ GPVI and integrin α_2_β_1_ receptors as well as the binding domain for the A3 domain of VWF. The peptides were mixed to a final concentration of 250 µg/ml each in 10 mm acetic acid, incubated for 2 hr at room temperature in a microfluidic channel (*l* = 49 mm, *w* = 100 µm, *h* = 50 µm), and rinsed with 0.1% (w/v) Texas Red in 10 mM acetic acid to locate the strip by fluorescence. A microfluidic device consisting of 32 parallel channels (*w* = 300 µm, *h* = 50 µm) was placed perpendicular to the strip of peptides. Channels were blocked with 2% bovine serum albumin in PBS pH 7.4 for 45 min. Mouse blood samples were incubated with DiOC6 in DMSO (final concentration 1 µM) at 37°C for 10 min. Blood was then added to reservoirs on the device and perfused for 5 min at 300 s^−1^ and 1,500 s^−1^ using a syringe pump (Harvard PhD Ultra) in withdraw mode. Four technical replicates were performed on each sample and each condition was repeated twice on separate days. Platelet accumulation was measured by fluorescent images captured in each channel using motorized stages on an inverted microscope (Olympus IX83—40X objective—NA 0.6).

### 2.4 Assessment of platelet activation by flow cytometry

Assessment of platelet activation by flow cytometry was performed by diluting washed platelets (1 × 10^6^ platelets/ml) in Tyrodes buffer containing 1 mM CaCl_2_. Murine platelets were activated with thrombin (0.1 IU/ml) in the presence of anti‐mouse CD41‐BV421 antibody (Biolegend, Clone # MWReg30; 1:50), anti αIIbβ3 in active conformation (Emfret; clone JON/A‐PE; 1:25), P‐selectin‐APC (Biolegend, clone APM‐1; 1:25), or bovine Lactadherin‐FITC (Haematologic Technologies; 10 µg/ml). The activation was quenched at 5 min using ice‐cold 1% PFA Tyrodes buffer. Samples were run in the Gallios analyzer (Beckman Coulter). Studies were performed with *n* = 3–5 mice/day per group and repeated at least twice. Flow cytometry data were analyzed using Kaluza flow analysis software (Beckman Coulter) and Flowjo (Flowjo, LLC). Gating strategy as previously described (Davizon‐Castillo et al., 2019 **).**

### 2.5 Measurement of 5‐HT and PF4 by ELISA

Platelet‐rich plasma and PPP samples were obtained as described above. Studies were performed with *n* = 8–19 mice and ELISAs were run on two separate days. Total lung homogenates were prepared in lysis buffer containing protease and phosphatase inhibitors. PF4 and 5‐HT levels were measured using mouse ELISA kits (Abcam, Cambridge, MA and GenWay Biotech, respectively) following the manufacturer's instructions.

### 2.6 Platelet 2A and SERT protein expression

Platelets were pooled from several mice from different litters to obtain 20 million platelets per sample and Western blot was performed on a single day with an n of 4–5 as previously described (Nozik‐Grayck et al., 2014). One sample from the bleomycin group was excluded from analysis as the result was 2 standard deviations outside of the mean. The following antibodies were used: 5HT 2A R (1:500, Santa Cruz), SERT (1:500, Abcam), and β‐actin (1:10,000, Sigma‐Aldrich). The same membrane was cut in two and probed separately for the 5‐HT 2A R and SERT. The full representative blots are shown in Figure 5. The 5‐HT 2A R antibody has been previously validated (Lofdahl et al., 2016) and the SERT antibody has been validated using a transfected cell line by the manufacturer. The species‐appropriate secondary IgG antibody was used (1:2,000, Millipore).

FACS: 15 µl of whole blood was incubated with 2 µl of CD41‐BV421 (MWReg30, 1:50, Biolegend), 5‐HT 2A R‐FITC (1:50, Abcam), and SERT‐PE (1:50, LS Bio) for 15 min at room temperature in the dark. Whole blood was then fixed and lysed using 500 µl 1‐step Fix/Lyse solution for 20 min. Cells were then analyzed on a Beckman Coulter Gallios flow cytometer. Platelets were defined by their overall low forward and side scatter along with positive staining for CD41‐BV421. After which, 2A and SERT expression was determined using shifts in the Mean Fluorescence Intensity. Flow studies were performed on 2 separate days for 5‐HT 2A R and a single day for SERT with an n of 4–7.

### 2.7 Lung platelet quantification

Histology and Immunohistochemistry: Lungs were flushed with PBS then inflation‐fixed at 25 cm H2O for 30 min with 4% paraformaldehyde for paraffin embedding. Immunohistochemistry was performed for CD41 using the rabbit polyclonal antibody (1:200, GTX113758, GeneTex) diluted in Dako Antibody Diluent (S0809 Agilent Dako). Sections were developed with Dako EnVision+ Dual Link System‐HRP (DAB+) (K4065 Agilent Dako) and counterstained with Light Green (STLGC100 American MasterTech Scientific).

FACS: 100 µl of a 1:10 dilution of CD41‐BV421 (Biolegend, Clone # MWReg30) in PBS was retro‐orbitally injected into the mice 5 min before collecting lungs to label intravascular platelets. Studies were performed on a single day with an n of 5–7. Lungs were homogenized as previously described (PMID 30024304). Whole lung digests were then stained with CD41‐APC (Clone # MWReg30, BD Biosciences) and CD42b‐FITC (Clone # Xia.G5, Emfret) to label whole lung platelets. Interstitial platelets were defined for positive staining for CD41‐APC and CD42b‐FITC but negative staining for CD41‐BV421 (CD41‐APC^Hi^, CD42b‐FITC^Hi^, CD41‐BV421^Lo^). Quantification was performed using 123eCount beads (Thermofisher) as previously described (Good et al., 2018).

### 2.8 Antibody validation

All antibodies used in this study have undergone validation in the course of this study or have been previously validated. Representative full‐length blots and details of antibody validation are presented in the methods.

### 2.9 Statistical analysis

Data were analyzed using Prism (GraphPad Software) by unpaired *t* test, or two‐tailed *t* test. Data were expressed as mean ± SE and significance defined as *p* < .05.

## 3 RESULTS

### 3.1 Bleomycin induces greater platelet accumulation in whole blood microfluidic flow assays

**Figure 1:**
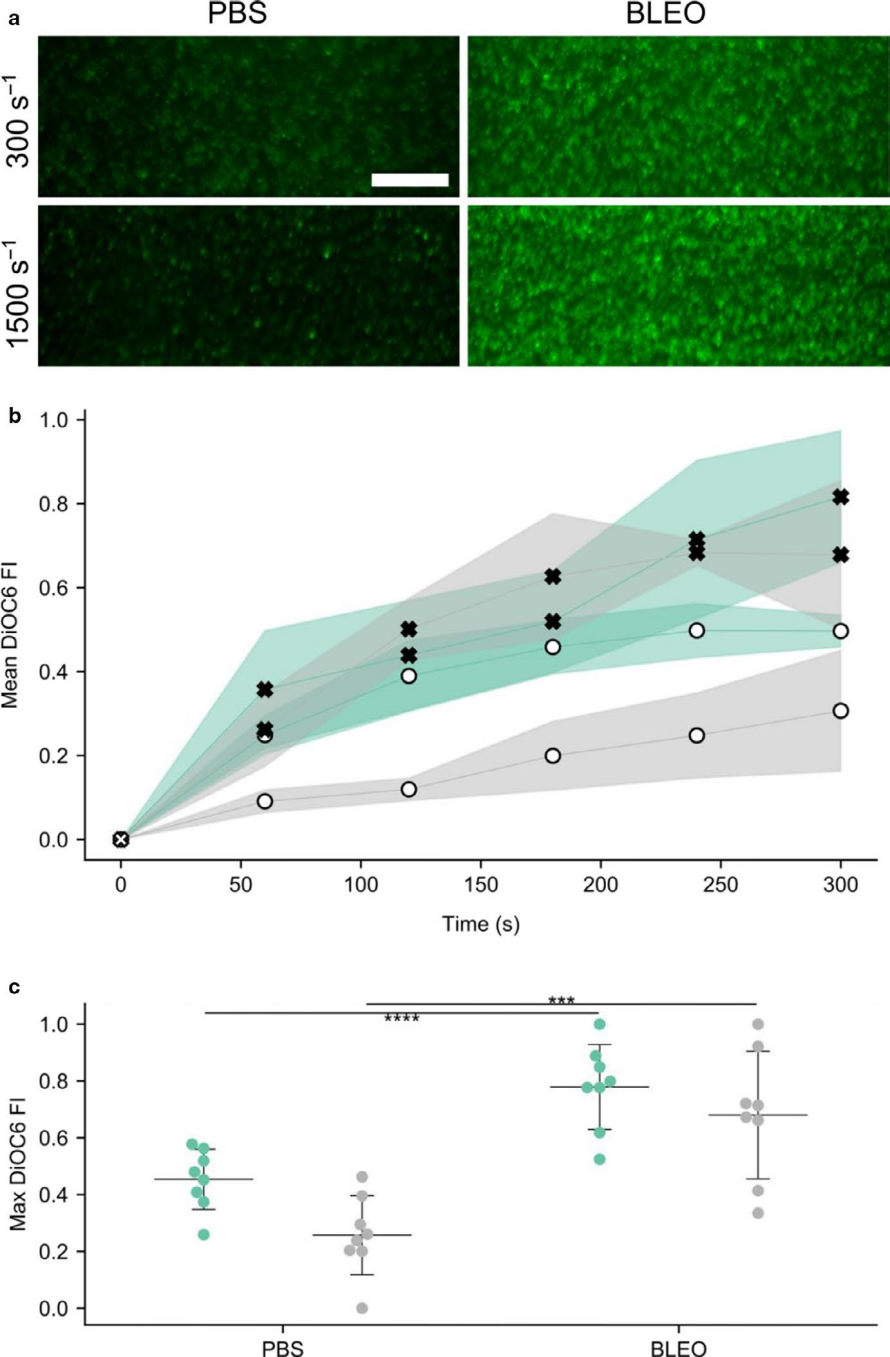
Platelets from mice with bleomycin‐induced PH demonstrate increased buildup during in vitro flow assays. Assays were run for 5 min at 300 s^−1^ and 1,500 s^−1^ to mimic venous and arterial shear rates. (a) Representative images of DiOC6‐labeled mouse platelets (scale bar = 50 µm) (b) Representative time series curves of platelet DiOC6 fluorescence. Green and grey regions depict assay shear rates of 300 s^−1^ and 1,500 s^−1^, respectively. Filled x's represent bleomycin‐treated mice, while open circles represent PBS treated mice. (c) Summary statistics of maximum DiOC6 FI for each assay. A single dot represents one assay, middle line shows the mean, and error bars display *SD*. At a given shear rate, blood from mice with experimental PH show an approximate twofold increase in platelet fluorescence. *** and **** denotes *p* < 10^−3^ and *p* < 10^−4^, respectively. Four technical replicates were performed on each sample and each condition was repeated twice on separate days, *n* = 4, PBS (1M, 1F), Bleo (1M, 1F), analysis by two‐tailed *t* test. PBS, phosphate‐buffered saline; PH, pulmonary hypertension

We performed whole blood microfluidic flow assays comparing platelets from mice treated with bleomycin to those treated with PBS. Blood was perfused over a patterned substrate of the collagen‐related peptides CRP, GFOGER, and VWF‐BP that mimic the functionality of type I collagen in terms of platelet adhesion, activation, and aggregation (Pugh et al., 2010). Assays were run for 5 min at 300 s^−1^ and 1,500 s^−1^ to mimic venous and arterial shear rates. At both shear rates, we observed an increase in platelet buildup in platelets from mice treated with bleomycin (Figure 1a). By quantifying the maximum fluorescence intensity of DiOC6‐labeled platelets, we saw an approximate twofold increase in platelet fluorescence in blood from mice with experimental PH and BPD (*p* < 1E‐3 for 1,500 s^−1^ and *p* < 1E‐4 for 300 s^−1^) (Figure 1b,c).

### 3.2 Platelet surface markers of activation are increased in neonatal murine PH

**Figure 2:**
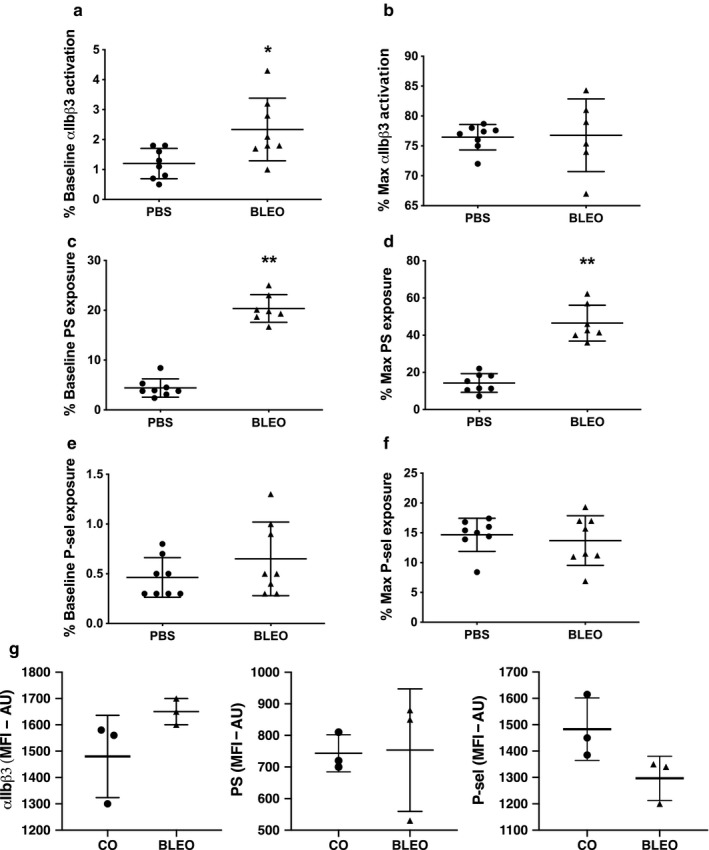
Platelets from mice are activated at baseline. (a) Platelets from mice with bleomycin‐induced PH have higher levels of active αIIbβ3 (main fibrinogen receptor) at baseline (unstimulated), *p < .05 by unpaired *t* test, *n* = 8 PBS (sex, not recorded), *n* = 8 Bleo (sex, not recorded). (b) Platelet activation after thrombin stimulation (0.1 IU/ml) for 5 min is similar between PBS and bleomycin. (c) Platelets from bleomycin‐induced PH exhibit significantly higher procoagulant potential by exposing higher amounts of phosphatidylserine on their surface at (c) baseline and after activation with (d) thrombin, **p < .0001 by unpaired *t* test, *n* = 7–8. Platelet degranulation as determined by measuring surface P‐selectin at (e) baseline and (f) after activation with thrombin are similar between groups, groups *n* = 8 PBS, *n* = 8 Bleo. (g) Incubation of pooled washed platelets from neonatal mice with comparable plasma concentrations of bleomycin does not lead to platelet activation of the αIIbβ3 integrin, PS or P‐selectin, *n* = 6 mice/group, pooled whole blood from 2 mice for each data point (4M, 2F). PBS, phosphate‐buffered saline; PH, pulmonary hypertension

The activation profile of washed platelets from mice with experimental PH showed a subtle but significant increase in baseline (circulating) activation of the αIIbβ3 integrin, the main fibrinogen receptor (Figure 2a). Platelets from PH mice and control mice exhibit similar active αIIbβ3 integrin on their surfaces after activation with thrombin (0.1 IU/ml) for 5 min (Figure 2b). In addition to exhibiting higher levels of active αIIbβ3 at baseline, platelets from PH mice have significantly higher levels of phosphatidylserine (PS), whose primary role is to provide a phospholipid platform for the assembly, activation, and amplification of the coagulation cascade in vivo. This difference is evident at baseline and upon activation with thrombin (Figure 2c,d). Despite these significant differences in phosphatidylserine and active αIIbβ3 integrin, we did not observe differences in P‐selectin at baseline or upon activation with thrombin (0.1 IU/ml) (Figure 2e,f). To determine whether bleomycin itself activates platelets, we incubated washed platelets with comparable plasma concentrations of bleomycin and found that bleomycin does not lead to platelet activation of the αIIbβ3 integrin or increased exposure of PS or P‐selectin (Figure 2g).

### 3.3 Plasma levels of platelet‐specific alpha and dense granule factors are increased in experimental neonatal PH

**Figure 3:**
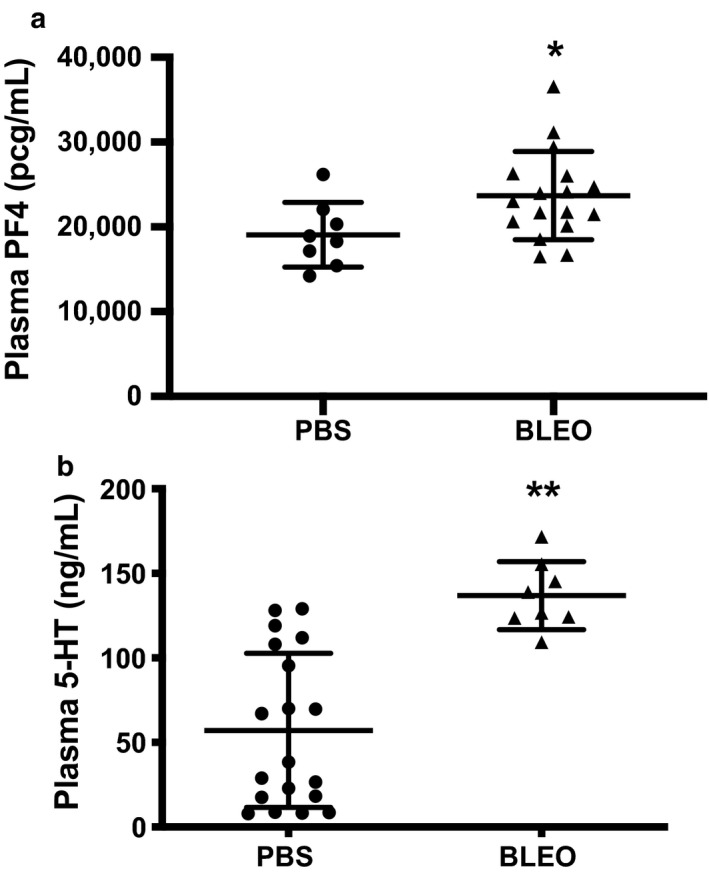
Plasma levels of platelet‐specific alpha (PF4) and dense granule (5‐HT) proteins are increased in experimental neonatal PH. (a) Platelet‐poor plasma PF4 levels from neonatal mice following IP PBS or bleomycin treatment, *p < .05 by unpaired *t* test, *n* = 8 PBS (6M,2F), *n* = 16 Bleo (9M, 7F). (b) Platelet‐poor plasma 5‐HT levels from neonatal mice following IP PBS or bleomycin treatment, **p < .0001 by unpaired *t* test, *n* = 8–19 (sex, not recorded). PBS, phosphate‐buffered saline; PF4, platelet factor 4; PH, pulmonary hypertension

To further assess whether experimental PH induces platelet activation, we measured PPP levels of the platelet‐specific alpha granule protein PF4 and the dense granule factor 5‐HT. In our murine model of bleomycin‐induced PH, platelet‐poor plasma levels of PF4 and 5‐HT are significantly elevated suggesting that baseline platelet activation of the αIIbβ3 integrin and PS exposure are accompanied with alpha and dense granule release (Figure 3a,b).

### 3.4 Platelet hematologic indices from mice with experimental PH are similar to controls

**Figure 4:**
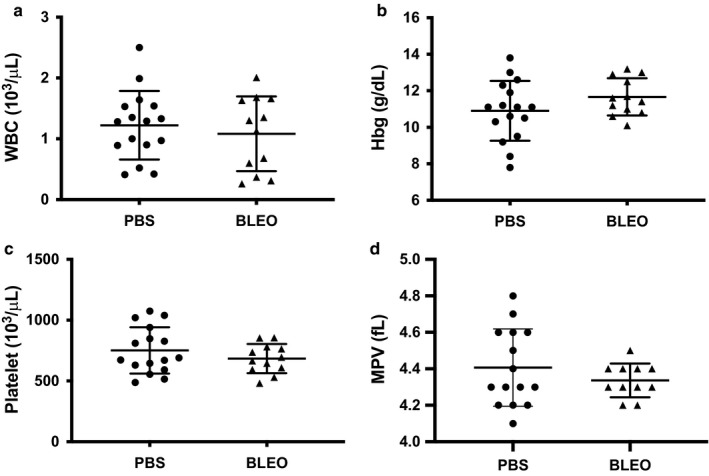
Platelet hematologic indices from mice with experimental PH are similar to controls. (a) Leukocyte counts in neonatal mice following IP PBS or bleomycin treatment, ns, *n* = 16 PBS (8M,8F), *n* = 11 Bleo (6M, 5F). (b) Hemoglobin levels in neonatal mice following IP PBS or bleomycin treatment, ns, *n* = 16 PBS (8M,8F), *n* = 11 Bleo (6M, 5F). (c) Platelet counts in neonatal mice following IP PBS or bleomycin treatment, ns, *n* = 16 PBS (8M,8F), *n* = 11 Bleo (6M, 5F). (d) Mean platelet volume (mpv) in neonatal mice following IP PBS or bleomycin treatment, ns, *n* = 15 PBS (7M, 8F), *n* = 10 Bleo (5M, 5F). PBS, phosphate‐buffered saline; PH, pulmonary hypertension

To determine whether bleomycin‐induced PH is associated with significant changes in bone marrow output, we obtained complete blood counts from control and PH mice. Total numbers of leukocytes, hemoglobin, platelets, and the mean platelet volume were not different between groups and suggests that bleomycin‐induced PH has no significant effect on bone marrow output of neonatal mice and that the increased platelet adhesion observed in the microfluidics assay is not due to the presence of higher platelet numbers in mice with PH (Figure 4a–d).

### 3.5 Platelet 5‐HT 2A receptor expression is increased in murine PH and BPD

**Figure 5:**
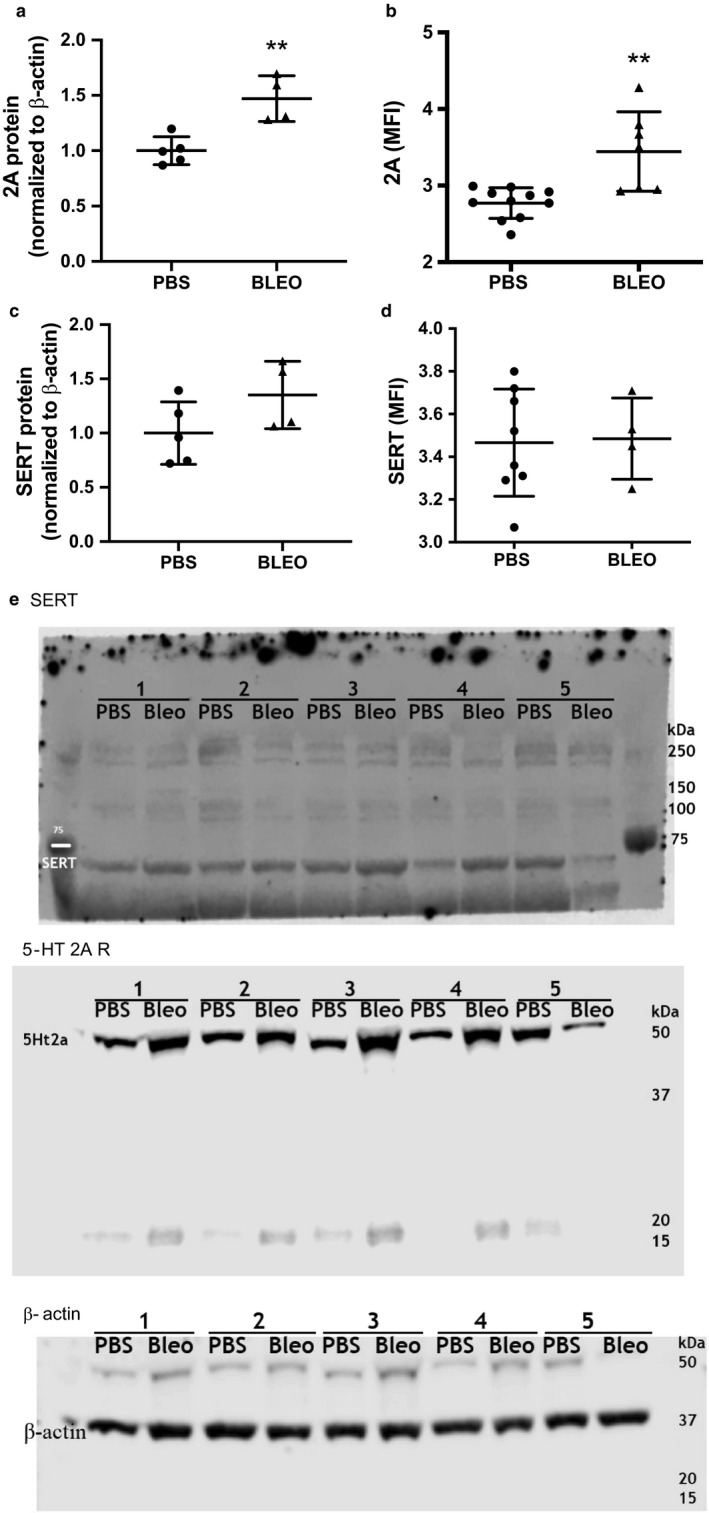
Western blot and FACS analysis for platelet 5‐HT 2A R and platelet SERT in mice treated with IP PBS or bleomycin. Bleomycin treatment increases platelet protein expression of the 5‐HT 2A R. (a) 5‐HT 2A R expression relative to β‐actin, **p < .005, by unpaired *t* test, *n* = 4–5 (pooled platelets from several mice, varied sex). One sample from the bleomycin group was excluded from analysis as the result was 2 standard deviations outside of the mean. (b) 5‐HT 2A receptor positive platelets in whole blood, **p < .005, by unpaired *t* test, *n* = 7 PBS (7M, 4F), *n* = 7 Bleo (4M, 7F). (c) SERT protein expression by relative to β‐actin, ns, *n* = 4–5 (pooled platelets from several mice, varied sex). One sample from the bleomycin group was excluded from analysis as the result was 2 standard deviations outside of the mean. (d) SERT‐positive platelets in whole blood, ns, *n* = 7 PBS (5M, 2F), *n* = 4 Bleo (2M, 2F). (e) Representative full‐length Western blots. PBS, phosphate‐buffered saline; SERT, serotonin transporter

The SERT is responsible for platelet 5‐HT uptake and the 5‐HT 2A R enhances local platelet aggregation and activation. After 3 weeks of treatment with either PBS or bleomycin, we analyzed protein expression of washed platelets by Western blot and platelets within whole blood by FACS to determine whether changes in 5‐HT 2A R and/or SERT were associated with the development of bleomycin‐induced neonatal PH. We found increased expression of the platelet 5‐HT 2A R protein by both Western blot of isolated platelets and FACs of circulating platelets (Figure 5a,b). Bleomycin‐induced PH did not change platelet SERT expression (Figure 5c,d).

### 3.6 Platelets are increased in the lungs of mice with experimental neonatal PH

**Figure 6:**
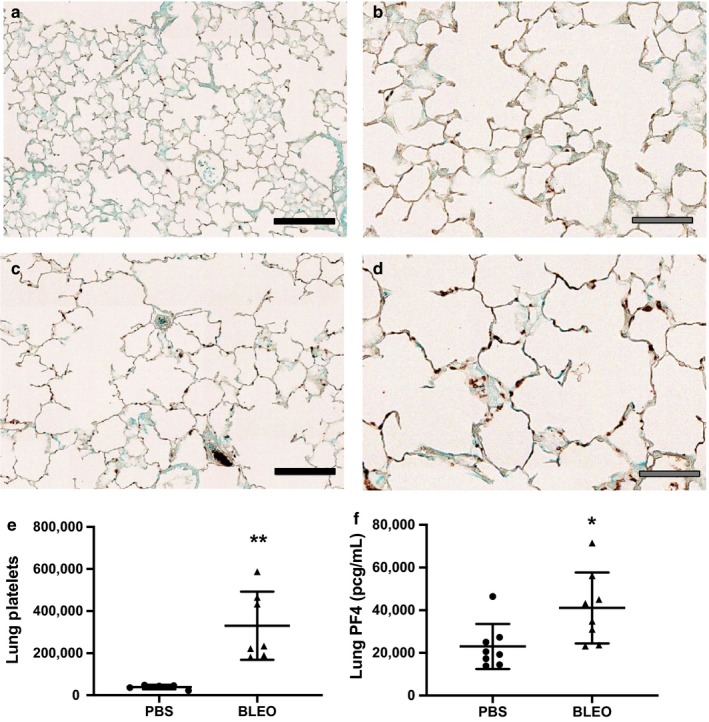
Platelets are increased in the lungs of mice with bleomycin‐induced PH. (a–d) Representative CD41 staining in 3‐week‐old mice treated with IP (a) PBS‐20 × magnification, (b) PBS‐40× magnification, (c) bleomycin‐20× magnification, or (d) bleomycin‐40× magnification, black filled scale bars = 100μm, grey filled scale bars = 50μm. (e) Interstitial platelets in neonatal mice following IP PBS or bleomycin treatment, **p < .005 by unpaired *t* test, *n* = 5 PBS (4M,1F), *n* = 7 Bleo (5M, 2F). (f) PF4 levels in whole lung homogenates from neonatal mice following IP PBS or bleomycin treatment, *p < .05 by unpaired *t* test, *n* = 8 PBS (6M, 2F), *n* = 8 Bleo (3M, 5F). PBS, phosphate‐buffered saline; PF4, platelet factor 4; PH, pulmonary hypertension

Lung sections of bleomycin‐treated mice showed increased amounts of platelets by immunohistochemistry (Figure 6a–d). Therefore, we quantitatively analyzed the absolute numbers of intravascular and interstitial platelets present in homogenized lung tissues using reference counting beads. Intravascular platelets were labeled with anti‐CD41‐BV421. We considered CD41‐APC/CD42b‐FITC double‐positive and CD41‐BV421‐negative platelets as interstitial platelets. We report that the number of interstitial platelets was significantly higher in lungs from bleomycin‐induced PH (Figure 6e). These results are in accordance with the elevated lung levels of the platelet‐specific protein, PF4 (Figure 6f).

## 4 DISCUSSION

We previously reported that pharmacologic blockade of the 5‐HT 2A R prevents bleomycin‐induced PH and pulmonary vascular remodeling (Delaney et al., 2018). As platelets contain 99% of circulating 5‐HT, which is released upon activation, we tested the hypothesis that circulating platelets are activated and increased in the lungs of neonatal mice with bleomycin‐induced PH. Through an extensive characterization of the functional status of platelets from bleomycin‐treated mice, we demonstrate that mice with bleomycin‐induced PH exhibit qualitative but not quantitative changes in circulating platelets. We show that circulating platelets from mice with PH exhibit a subtle but significant increase in platelet activation at baseline as evidenced by the higher percentage of circulating platelets with active αIIbβ3, the main platelet integrin involved in platelet aggregation. Moreover, we also show that these differences are functionally relevant as platelets from PH mice demonstrate greater accumulation than control littermates using our microfluidic assays. Similarly, significantly elevated plasma levels of the platelet‐specific proteins PF4 and 5‐HT further demonstrate higher baseline platelet activation in mice with PH. We also found that the absolute number of platelets within the lungs of mice with PH is significantly higher, altogether suggesting that platelets could directly be promoting PH. Ongoing work in our group focuses on the elucidation of the specific aspects of platelet function that directly favor the development of PH. Whether platelet adhesion to endothelial vasculature, platelet degranulation, transmigration, or platelet aggregation are required to promote PH remain to be studied.

Our data show that platelets from mice with PH appear to circulate in a “primed” state as evidenced by significantly higher levels of active αIIbβ3 and PS at baseline. Active αIIbβ3 has a high affinity for fibrinogen and fibrin and elevated baseline levels of active αIIbβ3 may favor platelet aggregation and adhesion to endothelial surfaces (Huang et al., 2019). Although the difference in baseline activation of the αIIbβ3 integrin is significant but subtle, higher baseline levels of active αIIbβ3 integrin detected by flow cytometry directly and functionally correlate with increased platelet accumulation on collagen. Upon platelet activation, platelets also expose PS. Exposed PS provides the phospholipid surface required for the assembly and amplification of the coagulation system. Therefore, the elevated amounts of PS on the surface of platelets from mice with PH in addition to the elevated levels of active αIIbβ3 are not only suggestive of higher platelet activation but also of increased procoagulant potential of platelets from mice with PH. Interestingly, recent work has shown that platelet PS can mediate the formation of neutrophil macroaggregates that promote pulmonary thrombosis in a model of murine intestinal ischemia/reperfusion (Yuan et al., 2017). Altogether, our results demonstrate that circulating platelets from mice with experimental PH have higher levels of active αIIbβ3 integrins that favor aggregation with other platelets and potentially to endothelial surfaces through immobilized fibrinogen or von Willebrand Factor (Kauskot & Hoylaerts, 2012). Ongoing work in our laboratory is aimed at investigating the mechanisms for increased platelet αIIbβ3 and PS observed in mice with PH and the functional significance as it relates to the pathogenesis of PH.

In addition to the functional platelet changes, we observed significantly higher levels of the platelet‐specific molecules PF4 and 5‐HT in the plasma of bleomycin‐treated mice suggesting ongoing alpha and dense granule release. As platelet activation is usually associated with release of all alpha granule contents, we were surprised by our results that despite observing higher plasma levels of the alpha granule, PF4, there is no difference in platelet expression of alpha granule, P‐selectin, between control and bleomycin‐induced PH mice. It is conceivable that this is due to previously described age‐related low expression and storage of P‐selectin in alpha granules of platelets from human neonates, murine fetal and neonatal platelets or due to neonatal agonist‐specific degranulation hypo‐responsiveness (Baker‐Groberg, Lattimore, Recht, McCarty, & Haley, 2016; Stolla et al., 2019).

Platelet‐derived PF4 inhibits endothelial cell proliferation in vitro and increased levels after birth are associated with higher rates of later pulmonary vascular disease in former preterm infants (Gengrinovitch, 1995; Maione., 1990; Wagner et al., 2018). 5‐HT release by activated platelets promotes pulmonary vasoconstriction, mitogenesis, and further platelet activation (Delaney, Gien, Grover, Roe, & Abman, 2011; Dunn, Lorch, & Sinha, 1989; Fanburg & Lee, 1997; Mammadova‐Bach et al., 2018; Walther et al., 2003; Yabanoglu et al., 2009). While higher levels of plasma PF4 and 5‐HT could reflect differences in clearance between control and bleomycin‐induced mice, it is more likely that these are the result of in vivo platelet degranulation as we observed a significantly higher pool of circulating activated platelets in bleomycin‐treated mice and no difference in SERT expression in platelets isolated from bleomycin‐treated mice. 5‐HT further enhances local platelet aggregation and activation via the platelet 5‐HT 2A R (McBride, 1990; Meuleman et al., 1983). We have previously reported that pharmacologic blockade of the 5‐HT 2A R with ketanserin prevents bleomycin‐induced PH and pulmonary vascular remodeling. Interestingly, we now show that platelets from mice treated with bleomycin demonstrate increased 5‐HT 2A R expression providing further evidence for platelet activation and serotonin signaling in this model and leads to the possibility that one mechanism for protection observed in ketanserin‐treated mice is blockade of platelet serotonin signaling.

A key finding of our study is the increased accumulation of platelets in the lungs of mice with experimental PH. This observation strongly suggests that platelets from bleomycin‐induced PH either adhere to the pulmonary vasculature or transmigrate into the perivascular space where they could directly deliver their granule contents. Increased lung intravascular and extravascular platelets have been reported in experimental models of acute lung injury, allergic lung inflammation, and sepsis (Cleary et al., 2019; Ortiz‐Munoz et al., 2014; Pitchford et al., 2008; Yuan et al., 2017). To our knowledge, this is the first report of increased extravascular lung platelets in a model of PH.

Our knowledge about platelet and megakaryocyte biology in neonates is relatively scarce. Findings from recent clinical studies suggest that elevated platelet counts at baseline in preterm infants and platelet transfusions may have deleterious effects on neonatal outcomes. High platelet counts after birth are an independent predictor of moderate and severe BPD and increased PF4 after birth is associated with increased rates of later pulmonary vascular disease in former preterm infants (Chen et al., 2019; Wagner et al., 2018). Preterm neonates randomized to receive platelet transfusions to maintain a higher baseline platelet count (50K) showed increased mortality when compared to preterm neonates with a lower transfusion threshold (25K) (Curley et al., 2019). Moreover, preterm neonates in the high transfusion baseline group had an increased incidence of BPD. Whether these complications are directly caused by platelets is difficult to establish; however, they illustrate the need to expand our knowledge on neonatal platelet biology and the interplay between platelets and neonatal lung disease.

There are a few potential limitations that warrant further investigation. While we measured plasma PF4 and 5‐HT as indicators of platelet activation, we recognize that platelets store hundreds of factors, including chemokines, growth factors, and vasoactive substances. Many of these factors have been implicated in the pathogenesis of neonatal PH promoting aberrant angiogenesis, increasing vascular tone, and inducing inflammation. However, it is conceivable to think that platelets could have a protective role in PH and BPD and future studies will focus on determining whether platelet activation is protective or promotes the development of neonatal PH. Another limitation of this study was the inability to definitely determine the source of increased lung platelets and the mechanisms leading to increased platelets within the lung including whether exposed collagen, fibrinogen, or VWF mediate increased platelet adherence. Our data demonstrate significantly higher numbers of platelets in the lungs of mice with PH; however, the number of circulating platelets remained comparable between control and PH mice. Given that the lung is a known site for extramedullary platelet biogenesis, it is possible that the increased number of lung platelets in mice with PH was due to higher lung megakaryocyte platelet production and not necessarily due to the recruitment of circulating platelets (Kaufman, Airo, Pollack, & Crosby, 1965; Lefrancais et al., 2017; Zucker‐Franklin, 2000). Future experiments utilizing transfusion of radiolabeled platelets will help address this question. Lastly, while our data demonstrate a clear association between platelet activation and neonatal PH, it remains unanswered whether platelets themselves or a secreted factor influence the phenotype of pulmonary vascular cells directly contributing to the pathogenesis of PH. Future studies by our laboratory will address these remaining questions.

In summary, we report that platelets are activated in neonatal murine PH in the setting of BPD, demonstrated by increased accumulation to collagen under physiologic flow conditions, increased platelet markers of activation, and increased plasma levels of alpha and dense granule stored factors. Our observations are in concordance with previously reported work in adults with PH where platelets exhibit increased in vivo activation and platelet hyperreactivity ex vivo, supporting our hypothesis that platelets are key players in the pathogenesis of PH (Maeda, Bydlowski, & Lopes, 2005; Nakonechnicov et al., 1996; Yaoita et al., 2014). In addition, we show that platelets from mice with experimental PH have increased expression of the 5‐HT 2A R which could further enhance local platelet aggregation and activation. Finally, we demonstrate that platelets are increased in the lung interstitium of mice with PH. We speculate that pharmacologic strategies targeting platelet activation in PH in the setting of BPD may provide a novel therapeutic strategy.
